# Morpho-Physiological and Genomic Evaluation of *Juglans* Species Reveals Regional Maladaptation to Cold Stress

**DOI:** 10.3389/fpls.2020.00229

**Published:** 2020-03-10

**Authors:** Aziz Ebrahimi, Shaneka S. Lawson, James R. McKenna, Douglass F. Jacobs

**Affiliations:** ^1^Hardwood Tree Improvement and Regeneration Center, Department of Forestry and Natural Resources, Purdue University, West Lafayette, IN, United States; ^2^USDA Forest Service, Northern Research Station, Hardwood Tree Improvement and Regeneration Center, West Lafayette, IN, United States

**Keywords:** cold hardiness, interspecific F1 hybrids, *Juglans*, signature of selection, Carpathian walnut

## Abstract

Climate change may have unpredictable effects on the cold hardiness of woody species planted outside of their range of origin. Extreme undulations in temperatures may exacerbate susceptibility to cold stress, thereby interfering with productivity and ecosystem functioning. *Juglans* L. and their naturally occurring interspecific F1 hybrids, are distributed natively across many temperate regions, and *J. regia* has been extensively introduced. Cold hardiness, an environmental and genetic factor yet to be evaluated in many native and introduced *Juglans* species, may be a limiting factor under future climate change and following species introductions. We evaluated cold hardiness of native North American and Eastern Asian *Juglans* along with *J. regia* genotypes using field data from the Midwestern United States (Indiana), controlled freezing tests, and genome sequencing with close assessment of *Juglans* cold hardy genes. Many *Juglans* species previously screened for cold-hardiness were genotypes derived from the Midwest, California, and Europe. In 2014, despite general climate adaptation, Midwestern winter temperatures of −30°C killed *J. regia* originating from California; however, naturalized Midwestern *J. regia* survived and displayed low damage. Hybridization of *J. regia* with black walnut (*J. nigra*) and butternut (*J. cinerea*) produced F1s displaying greater cold tolerance than pure *J. regia*. Cold hardiness and growth are variable in Midwestern *J. regia* compared to native *Juglans*, East Asian *Juglans*, and F1 hybrids. Phylogeny analyses revealed that *J. cinerea* sorted with East Asian species using the nuclear genome but with North American species using the organellar genome. Investigation of selected cold hardy genes revealed that *J. regia* was distinct from other species and exhibited less genetic diversity than native *Juglans* species Average whole genome heterozygosity and Tajima’s D for cold hardy genes was low within *J. regia* samples and significantly higher for hybrid as well as *J. nigra*. We confirmed that molecular and morpho-physiological data were highly correlated and thus can be used effectively to characterize cold hardiness in *Juglans* species. We conclude that the genetic diversity within local *J. regia* populations is low and additional germplasm is needed for development of more regionally adapted *J. regia* varieties.

## Introduction

Walnuts (*Juglans* spp.) occur around the world in a broad range of climates from Western and Eastern Asia to North, Central and South America (Ramos, 1997). Among *Juglans* species, Persian walnut (*Juglans regia* L.) is reported to have the most valuable edible nuts and high-quality timber. *J. regia* is found and grown worldwide, far from the Central Asian region where the species originated (Ramos, 1997; Guàrdia et al., 2013). In the Eastern and Midwestern United States, much of the introduced *J. regia* was derived from seeds collected in the Carpathian Mountain range during the mid-19th Century (Grimo, 1979). Seed collections from this eastern edge of Europe were designated “Carpathian walnut” and selected for tolerance to the cold temperatures found in Eastern and Midwestern North America (Ramos, 1997). Tracing the genetic origin of trees facilitates adaptive management for climate change (Aitken and Bemmels, 2016). Hamilton et al. (2013) evaluated the interaction of genotype with environment in spruce hybrids *(Picea sitchensis* × *P. glauca*) and noted that suitable genotypes could be identified for reforestation in areas where climatic conditions have changed or are predicted to change using growth and cold hardy traits.

Cold hardiness is a major limiting factor for *J. regia* cultivation and reforestation, particularly in the Midwest (Ebrahimi et al., 2017). *J. regia* has proven to be cold hardy but is not well adapted to the Eastern deciduous forests of the United States (Grimo, 1979; Pollegioni et al., 2017). Conditions such as winterkill, which destroys young branches, spring frost that damages flowers and new shoots, poor growth on acidic soils, and disease pressure given the higher humidity of North America relative to central Asia where the species originates are reoccurring issues (Manning, 1978; Grimo, 1979).

The greater environmental tolerance of interspecific hybrids, proven capacity to respond to variable climates, and quality timber production make them good candidates for reforestation efforts (Hamilton et al., 2013). Despite the known advantages of reforestation with adaptive hybrid species, the genetic background of interspecific F1 *Juglans* hybrids in the United States has not been determined. F1 hybrids of *J. regia* and *J. nigra* (*J. intermedia*) could be used for rootstocks to overcome the climate limitations and improve nut and timber production (McKay, 1965).

Cold hardiness is an important trait for abiotic stress adaptation but is difficult to track in the field, as the causative agent for injury or mortality can be difficult to pinpoint (Aitken and Bemmels, 2016). Native midwestern *Juglans* species (*J. nigra* and *J. cinerea*) can withstand cold temperatures to −40°C and -50°C, respectively (Weiser, 1970; Sakai and Weiser, 1973; George et al., 1977; Jacobs et al., 2008). Carpathian walnut is reportedly the most cold-hardy ecotype of *J. regia*, tolerating temperatures of −32 to −35°C without injury (Mitra et al., 1991). Researchers in France noted significant variability in cold hardiness among *J. regia* cultivars with a *J. intermedia* hybrid being the most tolerant (Poirier et al., 2004). Hardier than Carpathian walnut, *J. intermedia* endured −37°C temperatures with no damage (McKay, 1965; Grimo, 1979). Other factors associated with cold hardiness, such as carbohydrate content, also varied among species (Hyung et al., 2012).

Cold hardiness in woody plants may be evaluated in numerous ways, with visualization being the primary method for evaluating branches, buds, leaves, and twigs damaged by freezing temperatures (Sakai and Weiser, 1973; George et al., 1977). Cold hardiness can also be assessed using electrolyte leakage (McKay, 1992; Jacobs et al., 2008; Aslamarz et al., 2011), which occurs after cell death when loss of membrane integrity allows electrolytes, primarily potassium (K^+^), to cross the damaged membrane barrier unhindered. Quantification is obtained by measurement of increased electrolytic conductivity within the media containing dying cells (Lyons et al., 1979). Aslamarz et al. (2011) reported that levels of freezing damage for California *J. regia* cultivars were lower than Iranian cultivars. Sutinen et al. (1992) noted that visual and electrolyte leakage measurements of freezing damage in red pine (*Pinus resinosa*) were highly correlated. Patterson et al. (1976) suggested that measurement of electrolyte-leakage after chilling injury was much easier to evaluate than visible chilling injury.

Evaluation of cold hardy genes through qPCR is an inexpensive and reliable method for understanding gene expression in *Juglans* species. At present, no information regarding expression of cold hardy genes in *Juglans* species has been published. Numerous transcription factors have been found for evaluating freezing tolerance in woody plants (Benedict et al., 2006; Wisniewski et al., 2015). Dehydration-responsive element-binding proteins (DREBs) previously identified in *Arabidopsis* and woody plants were shown to play major roles in cold response (Gilmour et al., 1998; Wisniewski et al., 2015). Overexpression of a C-repeat binding factor (CBF) gene in apple (*Malus domestica*) resulted in delayed bud break in the spring and increased sensitivity to short photoperiods with respect to the start of dormancy (Wisniewski et al., 2015). Regulatory pathways in woody plants for cold hardy genes such as CBF are more complex than those of herbaceous plants as gene manipulation was shown to also affect growth and flowering (Benedict et al., 2006; Wisniewski et al., 2014).

Genome sequence technology can provide a greater depth of information for complex genes and pathways such as those involved in cold responses. A surge of interest in *Juglans* functional and comparative genomics was initiated after publication of a first draft of the *J. regia* genome in 2016 (Martínez-García et al., 2016). No genome sequence information linked to specific genes for cold hardiness and related traits has been published to date. However, chloroplast and genome-based data were used for phylogeny analyses of *Juglans* species (Hu et al., 2017; Stevens et al., 2018). Publication of a *Juglans* reference genome has provided researchers worldwide with the ability to make tremendous strides in a myriad of directions from additional phylogeny analyses and gene annotation to development of genetic linkage maps and gene filtering.

Statistical methods for analysis of nucleotide diversity and heterozygosity (i.e., Tajima’s D, π, F_*ST*_), highlight the lower allelic diversity in domesticated verses wild populations (Thornton and Jensen, 2007; Stevens et al., 2018). Cultivation of *J. regia* began in Persia 2000–2500 years ago (Potts, 2018). Human selection and domestication of *J. regia* for nut production could result in lower levels of genetic diversity in *J. regia* compared with wild relatives. Natural selection of *Juglans* species before their introduction to the United States may have led to population or diversity bottlenecks for adaptation to colder climates, such as *J. regia* and *J. ailantifolia*. Limited natural regeneration combined with genetic introgression of local populations after introduction of nut producing genotypes resulted in decreased wild *J. regia* population sizes (Demesure, 1996). Research by Clair (2006) indicated fewer cold hardy traits were expressed in selectively bred Douglas-fir (*Pseudotsuga menziesii*) than wild or unselected populations. A recent study based on whole genome data reported heterozygosity and genetic diversity within *J. regia* populations is lower than *J. nigra* and Texas walnut (*J. microcarpa*) (Stevens et al., 2018). These authors found that the polyphenol oxidase (PPO) gene was the least diverse within *J. regia* compared to *J. nigra* and *J. microcarpa*. Similar work in chestnut (*Castanea dentata*) reported a lack of variation in genes for nut traits for domesticated populations compared to wild populations (LaBonte et al., 2018).

Some *Juglans* traits, such as cold hardiness, have evolved over time and can now be used in selection studies (Ramos, 1997). Prevalence of cold periods and undulations in temperature in response to climate change have negatively impacted exotic *Juglans* species in the Midwest U.S. (Ebrahimi et al., 2017). Despite the many research studies in the literature on *Juglans*, none have focused on genetic mechanisms for cold hardiness. This work seeks to: (i) compare and contrast cold hardiness traits found in *J. regia* with those of North American *Juglans* species, Eastern Asia, F1 hybrids (crosses of cold hardy by exotic non-cold hardy) using both visual approximations and controlled freezing experiments; (ii) determine levels of cold hardy gene expression using qPCR; (iii) sequence and assemble 12 *Juglans* samples with paired end read data combined with whole genome and chloroplast phylogeny analyses of cold hardy genes; and (iv) identify signatures of selection for the cold hardy genes from various *Juglans* pools.

## Materials and Methods

### Plant Material

The Hardwood Tree Improvement and Regeneration Center (HTIRC)^1^ at Purdue University in West Lafayette, Indiana, USA established a germplasm collection with seven *Juglans* species and eight F1 hybrids in 2003 (Table 1 and Supplementary Appendix A) for its walnut timber breeding program. Those samples originated from the Midwestern United States, California, and Europe (Table 1 and Supplementary Appendix A). Between 1 to 3 grafted ramets or 1 to 6 half-sib seedlings were planted for each accession. An additional 26 *J. regia* samples were obtained from the Indiana Nut and Fruit Growers Association (INFGA)^2^ (Supplementary Appendix A) for use in growth and winterkill data collection. The INFGA trees ranged from 15–95 years old (35-year-old average) and were relatively well adapted to the Midwestern U.S. climate.

**TABLE 1:** List of *Juglans* and hybrid genotypes used in this study.

Cultivar/Genotype	Species	N^A^	Age^B^	Germplasm	Sources^C^
Butternut	*J. cinerea*	10	11	HTIRC	United States
Black walnut	*J. nigra*	10	11	HTIRC	United States
Buart	*J.* × *bixbi*	5	11	HTIRC	Indiana
Black × Persian walnut	*J.* × *intermedia*	2	10	HTIRC	Indiana
Persian walnut	*J. regia*	5	11	UC Davis	California
Persian walnut	*J. regia*	9	11	HTIRC	Midwest-United States
Persian walnut	*J. regia*	27	35	INFGA	Indiana
Manchurian walnut	*J. mandshurica*	2	10	HTIRC	Russia
Persian walnut × butternut	*J.* × *quadrangulata*	1	10	HTIRC	Indiana
Persian backcross^D^	*J. regia* × *[J. regia* × *J. nigra]*	1	11	HTIRC	Indiana
Japanese walnut	*J. ailantifolia*	5	10	HTIRC	California
Arizona walnut	*J. major*	10	11	HTIRC	Arizona
Black × Arizona walnut	*J. nigra* × *J. major*	1	10	HTIRC	Washington
Royal	*J. nigra* × *J. hindsii*	2	11	HTIRC	Missouri
Japanese × black walnut	*J. nigra* × *J. ailantifolia*	2	10	HTIRC	Indiana
N. Calif. black walnut^E^	*J. hindsii*	1	9	HTIRC	Illinois
Butternut backcross	*J. cinerea* × *[J. cinerea* × *J. ailantifolia]*	1	11	HTIRC	Indiana

*^*A*^Number of genotypes; ^*B*^Average age based on records and approximate estimates; ages in grafted genotypes are based on the tree rather than the ortet, ^*C*^Country or USA State where the accession originated to the best of our knowledge, ^*D*^This clone is an F_1_ backcross (J. regia × [J. regia × J. nigra]), ^*E*^Northern California black walnut.*

### Growth Data and Winterkill Ratings

**FIGURE 1:**
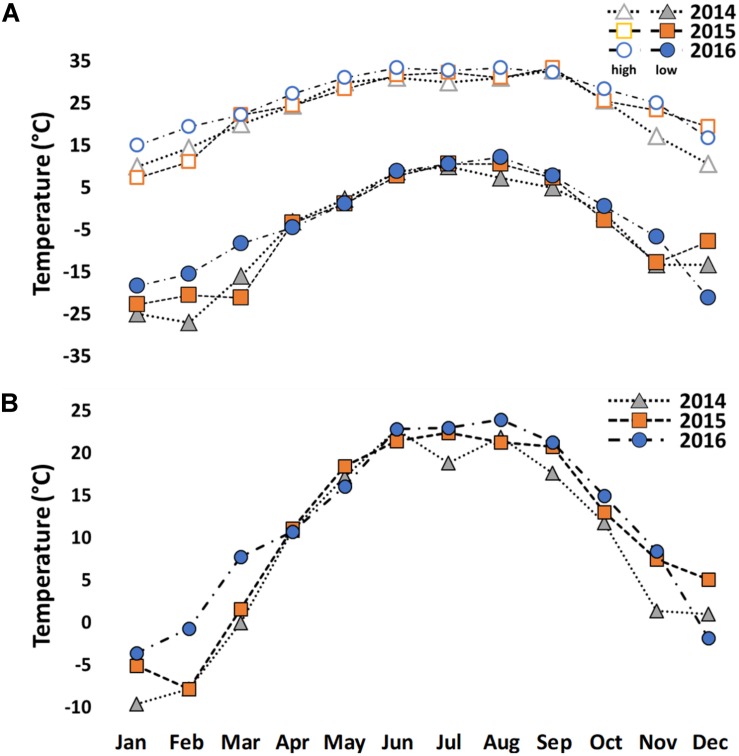
High and low temperatures **(A)** and average temperatures **(B)** from 2014 to 2016 in West Lafayette, IN, United States.

**FIGURE 2:**
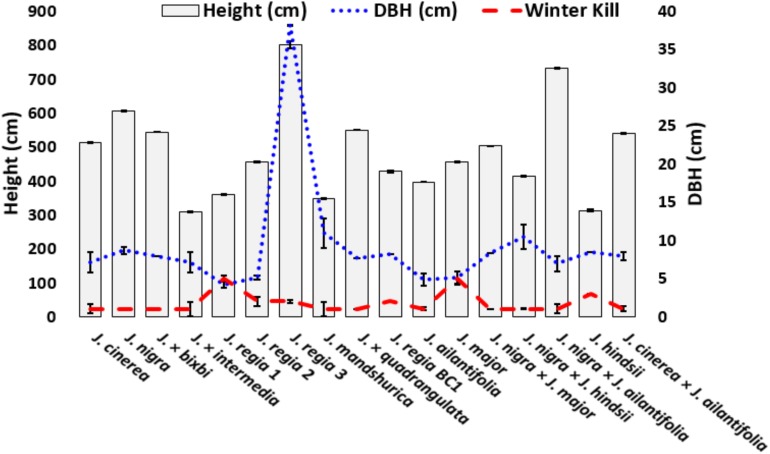
Mean (±SE) height and winterkill for studied *Juglans* species and interspecific hybrids. Heights were measured in winter 2014 and winterkill damage was measured in June 2014. *J. regia* 1 (California) and *J. major* (Arizona black walnut) were killed to the ground and did not re-sprout. Winterkill was rated from 1 to 5.

The winter of 2014 was unusually cold in Indiana (Figures 1A,B and Supplementary Appendix B). Study trees were measured for height and diameter at breast height (dbh) (Figure 2 and Supplementary Appendix C) in January 2014. Extremely low temperatures (−30°C) were recorded on two consecutive days in February 2014. Winterkill damage was assessed in mid-June 2014 after mild weather to allow for full symptom development from cold injury. Damage was qualitatively rated from 1 to 5 (1 = no visible damage; 2 = minimal twig damage (5–20%); 3 = moderate twig damage (20–50%); 4 = severe damage, dieback to ground with resprouting (50–90%); 5 = mortal injury, dieback to ground without resprouting (90–100%). For each biological replicate, 5-10 twigs were assessed for winterkill ratings. Regional weather data were obtained from the Bureau of Reclamation airport weather station in West Lafayette, IN, United States (Figure 1A,B and Supplementary Appendix B).

### Electrolyte Leakage

**FIGURE 3:**
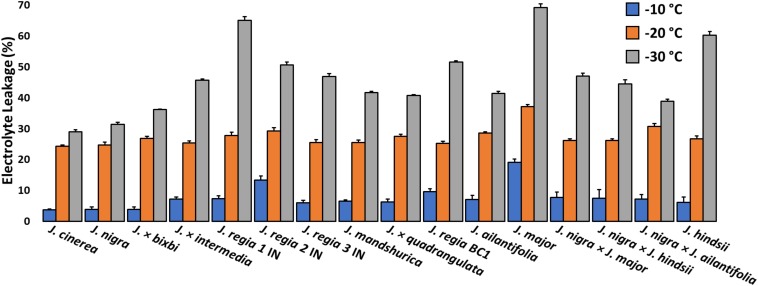
Index of freezing damage for 1-year-old twigs from *Juglans* species. and interspecific hybrids at –10, –20, and –30°C. Damage indices (D_*i*_) were obtained following protocols published by Earnshaw (1993). All *J. regia* used for electrolyte leakage analysis were obtained from Indiana and *J. regia* from California was excluded from this analysis. *J. major* was obtained from Indiana.

Electrolyte leakage (EL) was measured based on 3 to 6 one-year-old twigs for each genotype (Figure 3 and Supplementary Appendix D). Twigs were collected in January 2016 at the maximum of frost damage hardiness (Charrier et al., 2011). Several slices from each twig sample were weighed and examined for frost damage to glean a whole twig cold hardiness value (Charrier et al., 2013). EL was evaluated for three temperatures in a programmable freezer at −10, −20, and −30°C. Twigs were transferred into 20-ml tubes before being placed in each pre-set freezer. The initial temperature of the programmable freezer was 10°C, and the temperature was decreased at 0.30°C min^–1^. Temperatures were maintained for 1.5 h before being returned to room temperature (RT). Initial electrical conductivity (EC_*i*_) (Mettler Toledo, United States) was measured 24 h after control samples (4°C) were transferred to test tubes with 20 mL of distilled water. After freezing treatments, twig samples were returned to room temperature for 24 h before of EC_*i*_ was measured. All samples were autoclaved for 1 h at 100°C and final electrical conductivity (EC_*f*_) was assessed after the sample tubes cooled. Frost damage was approximated using relative frost index on (D*_*I*_*) values. Calculations of EL were based on the formula: D*_*I*_* = 100 (R_*t*_ – R_*o*_)/(1 – R_*o*_) where R_*t*_ = EC_*i*__/_EC_*f*_ of frozen samples and R_*o*_ = EC_*i*__/_EC_*f*_ of control samples (Earnshaw, 1993).

### RNA Extraction and qPCR

**FIGURE 4:**
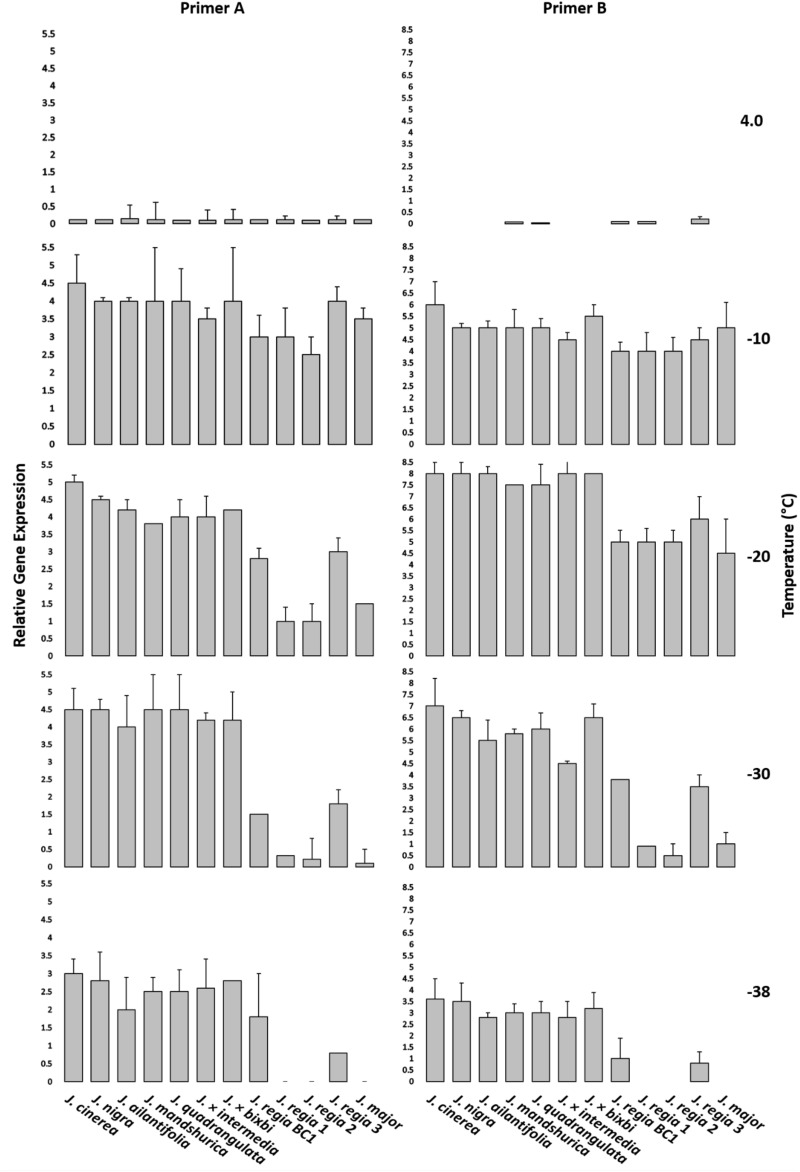
qPCR analysis. Two candidate primers for *Juglans* cold hardy genes were used to ascertain relative gene expression of twelve *Juglans* species and interspecific hybrid samples at 4, –10, –20, –30, and –38°C. Error bars represent standard error of the mean for three biological replicates (three technical replicates). All *J. regia* used for gene expression analysis were obtained from Indiana and *J. regia* from California was excluded from this analysis. *J. major* was obtained from Indiana.

Similar temperatures of EL test were used for gene expression analysis with the exception of −38°C that added to gene expression analysis. One of the subsamples was used to evaluate damage for EL, while the other was used for RNA analysis. Samples for RNA extraction were transferred to liquid nitrogen and stored at −80°C before isolation based on established protocols by Zarei et al. (2012). The qPCR template was generated from 1 μg total RNA used for First-strand cDNA synthesis with the Superscript III (Invitrogen, Carlsbad, CA, United States) kit. Primers for qPCR were designed with Primer3^3^ using the *J. regia* genome sequence data available in the NCBI database^4^. Actin was used as an internal qPCR control to measure gene expression. qPCR was performed using the Bio-RAD Real-time System (CFX connect, United States) with a SYBR Premix ExTaq Kit (Takara Bio, United States). Fold change in expression for the target cold hardy genes was based on the delta-delta Ct method (ΔΔCT) described in Livak and Schmittgen (2001). Three biological replicates (three technical replicates) for each sample were used for gene expression (Figure 4 and Supplementary Appendices E, F).

### Statistical Analyses

Field growth, electrolyte leakage, and gene expression data were analyzed using SAS software with PROC GLM mode (version 9.1; SAS Institute, Cary, NC, United States). When ANOVA was significant (*P* ≤ *0.05*), protected Fisher’s least significant difference (LSD) test (Steel and Torrie, 1980) was applied to describe differences between different *Juglans* species. All the statistical output results are listed in Supplementary Appendices C–F. The number of genotypes varied across *Juglans* species (Table 1), but for those with single genotypes at least 3-6 replicates were used for the traits examined.

### Genome Assembly and Sequencing

Analyses of field and electrolyte leakage data were made on 12 *Juglans* samples (Table 2). Genomic DNA was extracted from each sample following the protocol of Doyle and Doyle (1987). Samples were sequenced with 100 bp paired-end reads at Purdue Genomics Center^5^ using high throughput Illumina Hiseq 2500. Raw data were trimmed with Trimmomatic software (Bolger et al., 2014). Sequence fragments were assembled with SOAP-*de novo* software using trimmed reads using maximal read length = 100, average insert size = 400, and cutoff of pair number = 3 (Luo et al., 2012). Paired-end reads were mapped to the *J. regia* reference genome (Marrano et al., 2019) using the Burrow-Wheeler Aligner (BWA), Picard-tools (Langmead et al., 2009; McKenna et al., 2010; Wysoker et al., 2013) and SNP calling with the HaplotypeCaller tool from Genome Analysis Tool-kit (GATK) (McKenna et al., 2010). Consensus genome extracted with VCF tools and SAMtools. The assembled whole genome was used for whole gene prediction running AUGUSTUS (Stanke et al., 2006). A similar approach was used to assemble the chloroplast genomes of *Juglans* samples with *J. regia* chloroplasts used as reference (Martínez-García et al., 2016).

**TABLE 2:** Accession number, species, total sequence reads and assembly statistics for the 12 *Juglans* species used for evaluation of cold hardiness genes.

Accession No.	Species	Total sequence (million reads)	SN	TSL (bp)	N50
693	*J. regia* 1 IN	82	1944	2497141	1171
694	*J. regia* 2 IN	91	370642	299088527	500
Behr	*J. regia* 3 IN	42	219882	264763241	778
863	*J. regia* BC1^†^	40	166761	223992909	454
654	*J.* × *intermedia*	5	5537	4900133	119
208	*J.* × *quadrangulata*	67	61674	25578550	124
-	*J. major*	48	179585	140776978	545
OS-20	*J. cinerea*	73	148323	349509405	517
123 Rossville	*J.* × *bixbi*	38	237732	220543430	698
Purdue 1*	*J. nigra*	131	233964	379136826	119
910	*J. mandshurica*	101	193923	394316427	1419
1098	*J. ailantifolia*	61	188950	309942050	1250

SN, scaffold number; TSL, total scaffold length; N50, minimum contig length needed to cover 50% of the genome. *, well-known Midwestern cultivar of *J. nigra*; ^†^, 3/4 *J. regia* × 1/4 *J. nigra*. All the *J. regia* obtained from Midwestern Indiana, United States. *J. major* obtained from Indiana, United States.

### Phylogeny Analyses

**FIGURE 5:**
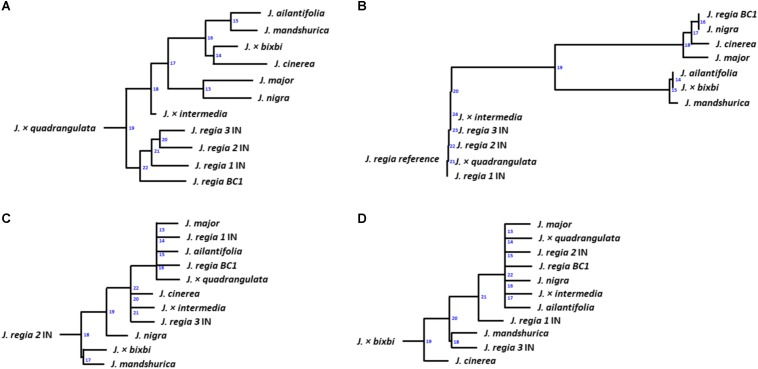
Dendrograms for samples of 12 *Juglans*: **(A)** based on whole nuclear genome; **(B)** based on chloroplast genome **(C)** based on whole proteins; **(D)** based on DREB-like genes filtered from the whole genome annotation. Numbers on transfers indicate the order of inference (Boc et al., 2012) for *Juglans* species.

Phylogenetic trees were created based on whole genome (paired-end reads) data using the Assembly and Alignment-Free (AAF)^6^ method described in Fan et al. (2015). The assembled chloroplast genomes were aligned with Molecular Evolutionary Genetic Analysis (MEGA) software (Tamura et al., 2007) to obtain a similarity/distance matrix for phylogeny analysis. After whole-genome annotation, cold hardiness-related proteins predicted by AUGUSTUS software in .gff (general feature format) format were converted to .fasta format with perl script. Each .fasta file of predicted proteins was subjected to Blastp analysis (Buchfink et al., 2015) that UniProt^7^ database used as a reference. Hits obtained from Blastp were averaged and the information used to create a similarity matrix. Genetic relatedness among *Juglans* samples was calculated by maximum likelihood following Tamura et al. (2007). A distance tree (Figure 5) was created with T-REX web server (Boc et al., 2012).

### Identification of Cold Hardy Genes

Evaluation of cold hardy genes predicted by AUGUSTUS was done following the protocol of LaBonte et al. (2018). The predicted genes were aligned to the UniProt^7^ database using DIAMOND sequence aligner on Blastp default mode using cold hardy gene keywords DREBs-like proteins (Buchfink et al., 2015). Genes identified after the initial blast search were again blasted against the UniProt protein database with DIAMOND sequence aligner on Blastp mode. Putative gene function was assigned based on of the top hits blasted.

### Cold Hardy Genes Based on Signature of Selection

In addition to 12 *Juglans* samples described in Table 2, we downloaded 26 *Juglans* genomes previously deposited in the NCBI database (Bai et al., 2018; Stevens et al., 2018). Those samples contain five *J. nigra*, eight *J. regia* from Asia and United States, four *J. mandshurica*, four *J. ailantifolia*, four *J. cinerea*, two *J. major*. We used nine pools for calculation of population indices designated as Japanese walnut (*J. ailantifolia*); Black walnut (*J. nigra*); Butternut (*J. cinerea*); Hybrid (interspecific F1s); Arizona walnut (*J. major*); Manchurian walnut (*J. mandshurica*) and Persian walnut (*J. regia* from Asia and United States considered as a separate pools). Despite being composed of a variety of species, all hybrids (Table 2) were pooled together (*J.* × *bixbi*, *J.* × *intermedia*, *J.* × *quadrangulata*, and a BC1 [*J. regia* × [*J.* × *intermedia*]). Tajima’s *D*, heterozygosity (Het), SNP variation, nucleotide diversity (π), and *F*_*ST*_ for a nine pooled genome for cold hardy gene were calculated with VCF-tools^8^ and perl scripts obtained from LaBonte et al. (2018). A negative Tajima’s *D* value and low values for π and proportion of heterozygous loci for a predicted gene among *Juglans* samples was considered evidence for signature of selection (Table 3 and Supplementary Table 1). Additional information regarding selective sweep or bottleneck regions was obtained by calculating Tajima’s *D*, nucleotide diversity (π), *F*_*ST*_ and SNP variation for genes chosen at random across the whole genome for approving cold hardy gene (Supplementary Table 2).

**TABLE 3:** Statistical data of population indices for cold hardy genes among the nine identified *Juglans* pools.

Chromosome	Gene	Gene function	Tajima’s D Analysis								
			JW	BW	BN	HY	AW	MW	PWA	PWU	FST
Chr01	g129	DRE1B_ARATH	–0.05	–0.168	–0.21	1.08	0.623	1.039	–0.72	nan	0.807
Chr01	g251	DREB3_ARATH	0.470	0.213	–0.48	0.00	0.372	0.593	1.086	–1.11	0.641
Chr01	g303	DREB3_ARATH	–0.10	–0.138	0.659	0.967	1.633	0.839	1.223	–0.14	0.771
Chr01	g304	DREB3_ARATH	–0.65	NA	–0.16	0.86	0.650	–0.57	0.328	0.298	0.464
Chr01	g308	ERF12_ARATH	0.919	1.059	nan	0.799	nan	0.722	0.238	–1.354	0.584
Chr01	g350	ZAT10_ARATH	0.390	–0.132	0.461	1.866	nan	–0.178	1.676	–0.214	0.690
Chr01	g371	ERF80_ARATH	0.603	0.298	0.396	0.976	nan	0.772	–0.510	nan	0.716
Chr03	g12997	DRE1B_ARATH	NA	–1.160	nan	1.173	nan	–1.199	nan	NA	0.812
Chr03	g13333	DRE2D_ARATH	–0.00	–0.278	0.756	0.683	NA	0.985	–1.00	–0.896	0.682
Chr04	g18519	EF103_ARATH	–0.07	nan	nan	1.094	nan	–0.400	–0.164	nan	0.776
Chr05	g24025	DREB3_ARATH	nan	nan	nan	1.413	nan	0.088	nan	nan	0.767
Chr05	g24084	ZAT10_ARATH	nan	nan	nan	1.302	nan	nan	nan	NA	0.751
Chr05	g24112	ERF80_ARATH	–0.68	–0.180	–1.04	1.652	0.392	–1.311	0.486	0.852	0.730
Chr06	g27449	ERF80_ARATH	0.542	–0.932	nan	nan	–0.162	nan	0.233	nan	0.800
Chr07	g33652	ERF80_ARATH	0.338	–0.625	–0.54	1.704	0.372	0.730	–1.099	NA	0.840
Chr08	g41530	DREB3_ARATH	–1.05	–0.617	–0.41	1.285	0.031	–1.310	0.881	–0.800	0.767
Chr08	g41803	DREB3_ARATH	1.325	0.361	nan	1.335	nan	0.113	nan	nan	0.856
Chr08	g41807	ICE1_ARATH	0.138	–0.739	0.499	1.742	1.112	0.704	–0.187	–0.709	0.856
Chr09	g46244	DREB3_ARATH	–0.53	–0.401	nan	0.832	nan	0.051	–0.547	–0.363	0.736
Chr10	g49957	DREB3_ARATH	0.354	0.030	0.709	0.902	0.123	0.463	–0.037	–0.084	0.787
Chr10	g49981	DREB3_ARATH	–0.05	nan	1.435	nan	nan	nan	0.492	–0.028	0.602
Chr10	g49983	DREB3_ARATH	0.418	nan	nan	1.340	nan	–0.382	0.425	0.541	0.700
Chr10	g49988	ZAT10_ARATH	–0.81	–0.715	0.650	1.210	0.467	–1.055	–1.141	NA	0.854
Chr10	g50022	ERF80_ARATH	0.682	0.588	–0.33	1.560	0.376	0.899	–0.549	0.021	0.793
Chr10	g50035	EF2_ARATH	0.995	1.953	0.214	1.139	NA	0.270	1.005	0.139	0.705
Chr11	g56121	ERF80_ARATH	0.253	–1.113	0.868	1.593	0.454	0.610	–0.155	nan	0.846
Chr13	g66409	ZAT10_ARATH	–1.43	–0.383	nan	0.211	nan	0.197	–1.582	nan	0.806
Chr13	g66538	EF103_ARATH	nan	nan	nan	1.161	nan	nan	0.335	0.452	0.816
Chr14	g72786	EF103_ARATH	nan	1.316	0.015	1.524	1.331	0.242	nan	nan	0.505
Chr14	g72790	DREB3_ARATH	nan	nan	nan	1.182	nan	0.290	nan	nan	0.806
Chr14	g72833	ZAT10_ARATH	0.843	0.091	nan	nan	nan	–0.057	nan	NA	0.766
Chr14	g72872	ZAT4_ARATH	0.039	0.251	–0.61	0.647	nan	0.685	–0.493	–0.638	0.727

*JW, Japanese walnut; BW, Black walnut; BN, Butternut; HY, Hybrid; AW, Arizona walnut; MW, Manchurian walnut; PWA, Persian walnut Asia; PWU, Persian walnut United States; FST, Total genetic variance using selected gene interval.*

## Results

### Field Ratings

*Juglans* species exhibited a wide range of growth differences in response to cold temperatures (Figure 2 and Supplementary Appendix C). Timber stem quality of *J. regia* was average at best and most commonly appeared below average to poor. *J. regia* exhibited a reduction in growth compared to cold hardy *J. cinerea* and *J. nigra* (Figure 2 and Supplementary Appendix C). The *J. regia* BC1 ([*J. nigra* × *J. regia*] × *J. regia*)] grew comparable to *J. cinerea* and *J. nigra* as did the F1 hybrid *J.* × *quadrangulata* (*J. regia* × *J. cinerea*). Hybrids ‘Royal’ (*J. hindsii* × *J. nigra*) and *J.* × *bixbyi* (*J. ailantifolia* × *J. cinerea*) had greater growth than *J. cinerea* and *J. nigra*. Average dbh among *Juglans* samples varied from 4.2 cm (*J. regia* CA) to > 10.9 cm for Manchurian walnut (*J. mandshurica*) (Figure 2 and Supplementary Appendix A). The dbh values for native *Juglans* species (*J. cinerea*, *J. nigra*) and their F1 hybrids were all greater than that of *J. regia* originally from California and the Midwest U.S (Figure 2 and Supplementary Appendix C).

Very little cold damage was observed in the *Juglans* Germplasm block from 2003–2013. Damage occurred presumably in February 2014 after low temperatures dropped to a low of −30°C for about 4 h two consecutive days (Figures 1A,B). As a result, all *J. regia* had various levels of winterkill ranging from slight damage for Carpathian (*J. regia* IN 3) to 100% mortality for Mediterranean varieties (*J. regia* California) which were killed entirely to their graft unions (Figure 2 and Supplementary Appendix C). Arizona walnut (*J. major*), comprised of seven high-elevation half-sib families from Arizona U.S. displayed winterkill damage similar to *J. regia*. When cold-sensitive *Juglans* species. (*J. hindsii*, *J. major, J. regia*) were hybridized with cold hardy *Juglans* species. (*J. cinerea*, *J. nigra*), the resultant F1 hybrids exhibited tolerance akin to the pure cold hardy species (Figure 2 and Supplementary Appendix C). Like *J. cinerea* and *J. nigra*, Japanese walnut (*J. ailantifolia*) and Chinese walnut (*J. mandshurica*) showed little winterkill (Figure 2 and Supplementary Appendix C).

### Cold-Hardiness Evaluation After Controlled Freezing

All of our *J. regia* from California perished in 2014 after exposure to unusually cold (−30°C) winter temperatures, and so this source was excluded from the controlled-freezing tests. A comparison of cold damage indices among the remaining *Juglans* species revealed that California black walnut (*J. hindsii*), *J. major*, and *J. regia* had significantly greater damage (*p* < 0.01) than other *Juglans* species (Figure 3 and Supplementary Appendix D). The two *Juglans* species that showed the least damage were *J. cinerea* and *J. nigra*. East Asian species (*J. ailantifolia* and *J. mandshurica*) had less damage than *J. regia* and F1 hybrids but more damage than *J. cinerea* and *J. nigra*. When less hardy *Juglans* species were crossed with hardier *Juglans* species, cold hardiness of the offspring significantly increased from previous levels. While cold damage was lower in F1 hybrids but not to the level of pure cold hardy species (Figure 3 and Supplementary Appendix D).

### Cold Hardy Gene Expression

Two primers indicated significant variation in the freezing response for *Juglans* species. (Figure 4 and Supplementary Appendices E, F). *J. cinerea* and *J. nigra* had higher gene expression for all examined temperatures. Cold hardy genes were highly expressed in *J. regia* (a non-cold hardy species) at −10 and −20°C but levels decreased dramatically at lower temperatures (−30 and −38°C). Gene expression levels for F1 hybrids (*J. regia* × cold hardy species) were higher than for pure *J. regia.* East Asian species (*J. ailantifolia* and *J. mandshurica*) had lower gene expression compared with *J. cinerea* and *J. nigra* but displayed gene expression levels like F1 hybrids. *J. regia* had the lowest gene expression of the *Juglans* species sampled.

### Phylogeny Analysis

Twelve *Juglans* samples were evaluated based on their chloroplast, whole-genome, protein, and DREB-like gene similarity (Table 2). Phylogeny analyses of whole genome data indicated that the *Juglans* samples sorted into three main groups based on their continent of origin. However, the North American *J. cinerea* sorted with East Asian species (Figure 5A). F1 hybrids sorted between the groups containing their parents (Figure 5A). Phylogeny analyses generated from the chloroplast data revealed a similar sorting pattern although *J. cinerea* now sorted with North American *Juglans* species and each hybrid sorted with their maternal parent (Figure 5B). Analyses of protein data revealed an inconsistency as the categorization of *Juglans* changed both within and among species (Figure 5C). For example, each of the three cold hardy *J. regia* samples from Indiana were placed into different groups. Phylogeny analyses created from DREB-like genes showed *J. cinerea* and *J.* × *bixbi*, the most cold hardy *Juglans* species sampled, sorted into separate groups but *J. nigra*, also cold hardy, sorted with the F1 hybrid samples (Figure 5D).

### *Juglans* Genome Signature of Selection

Selective sweeps within cold hardy genes were evaluated within the nine *Juglans* pools based on heterozygosity and Tajima’s *D* analyses. Thirty-three cold hardy genes with various Tajima’s *D* in all *Juglans* pools were chosen for evidence of selective sweeps (Table 3 and Supplementary Table 1). Numerous intervals with elevated variation in Tajima’s *D* among different pools were selected for further gene annotation analysis and concomitantly, these regions with lower Tajima’s *D* also display lower genetic diversity. The average value of Tajima’s *D* for cold hardy genes among the *Juglans* species varied from -0.237 (*J. regia* from United States) to 1.146 (Hybrid) (Table 3 and Supplementary Table 1). Tajima’s *D* values for most of the cold hardy genes in hybrid samples were ≥1, a rare occurrence for other *Juglans* pools (Table 3 and Supplementary Table 1). Heterozygosity of cold hardy genes in the different *Juglans* species undulated from 0.0074 (Persian walnut United States) to 0.0264 (F1 hybrids) and 0.0216 (black walnut) (Table 3 and Supplementary Table 1). Analysis of the selected cold hardy genes revealed 1794 SNPs that varied across pools with Persian walnut from Asia (176, lowest) to Manchurian walnut from China (250, highest) (Supplementary Table 1). The cold hardy genes in this study showed unusually low heterozygosity and nucleotide diversity within the nine pools of *Juglans* species; however, average heterozygosity (*Het*_*avg*_) was significantly lower for *J. regia* than for hybrids and black walnut. The whole genomes from each pool of *Juglans* species also were investigated for presence of signature of selection for random gene across chromosome one. Average random genome values for Tajima’s *D* were −0.0457 (Persian walnut, from Asia), to 0.782 (interspecific F1 hybrids), 0.0095 (*J. cinerea*, butternut), and −0.114 (*J. nigra*, black walnut) (Supplementary Table 2). Random genome heterozygosity varied from 0.01 in *J. regia* to 0.046 (F1 hybrids), 0.015 in butternut and 0.023 in black walnut (Supplementary Table 2). Assessment of genes chosen at random confirmed that *J. regia* exhibited the least diversity of all *Juglans* species studied (Supplementary Table 2), a result validated by a selective sweep of cold hardy genes.

## Discussion

### Field Ratings

Assessments of cold hardiness and growth for local and introduced *Juglans* species are vital for the success of *Juglans* plantations. In this study, native species (*J. cinerea* and *J. nigra*) and their F1 hybrids grew faster and were hardier than *J. regia* species. Growth of *J. regia* was poor compared to related F1 hybrids and native *Juglans* species (*J. cinerea, J. nigra*); however, growth of the *J. regia* BC1 (*J. regia* × [*J. nigra* × *J. regia*]) was comparable to pure *J. cinerea* and *J. nigra*. Overall, our data indicate a significant correlation between cold hardiness and growth and survival. Cold tolerant hybrids grew at rates similar to native cold hardy species and had improved timber form. Cold hardiness among *J. regia* selections varied by origin, with Carpathian varieties being much more cold hardy than Mediterranean varieties, from France and California (‘Franquette’, ‘Fernette’, and Fernor’) being not cold hardy (Figure 2 and Supplementary Appendix A). Severe cold temperatures of 2014 caused some damage to even our hardiest *J. regia* IN 3, ‘Behr,’ which was nearly 60 years old.

Guàrdia et al. (2013) evaluated cold hardiness in various *Juglans* species and rated *J. nigra* as most cold hardy and *J. regia* as least. In addition, they found that *J. nigra* acclimated quicker to cold than *J. regia* (Guàrdia et al., 2013). Cold hardiness of the F1 hybrid, *J.* × *intermedia* was intermediate to that of pure species (Mitra et al., 1991). Our findings confirmed that (cold-sensitive × cold-tolerant) hybrids inherit a combination of cold genes from each parent, and that cold hardiness is a genetic and highly heritable trait. Such interspecific hybrids show the potential for assisted migration of pure ecotypes where cold tolerant northern populations could be crossed with cold sensitive but heat tolerant populations (Hamilton et al., 2013). Natural hybrids can be genetically diverse and well suited to compete with conspecifics (Aitken and Bemmels, 2016).

### Controlled Freezing

Electrolyte leakage (EL) results indicated that *J. regia* IN, *J. major*, and northern California black walnut (*J. hindsii*) had significantly greater damage indices (*p* < *0.01*) than native species *J. cinerea, J. nigra* and F1 hybrids at −30°C (Figure 3 and Supplementary Appendix D). Research by Guàrdia et al. (2013) reported that *J. nigra* was hardier than *J. intermedia* and *J. regia*; and Aslamarz et al. (2011) found that California-based *J. regia* cultivars were hardier than Iranian cultivars. Patterson et al. (1976) examined different *Passiflora* spp. from diverse climates using EL and noted tropical lowland species were more-susceptible than those originating in colder climates. Many methods for evaluation of cold hardiness exist, but measurement of electrolyte leakage cell death is less difficult and more sensitive than visual observations of injury (Patterson et al., 1976). Laboratory estimates of cold tolerance and those from data collected in the field tend to be highly correlated. Laboratory estimates may be slightly higher compared to damage under field conditions (Schaberg and DeHayes, 2000), and our own observations. However, the ease and speed of screening material through EL is compelling for breeding and forest management.

### Gene Expression Through qPCR

In addition to EL tests, gene expression of two putative cold hardy genes was investigated using qPCR (Figure 4 and Supplementary Appendices E, F). Close inspection of gene expression data indicated that cold hardy gene expression patterns mirrored results seen in the field as well as EL data. Cold-susceptible species such as *J. regia* showed little to no gene expression at −30 and −38°C, a plausible explanation for the increased mortality in winter 2014. Although one *J. regia* (*J. regia* 3 IN) had greater expression than the other *J. regia*, expression levels were much lower than for native *Juglans* species and F1 hybrids. Cold hardiness in relation to genetic composition of *Juglans* species was corroborated when F1 hybrids with *J. regia* parents were examined; F1 hybrids with a greater composition of *J. regia* had lower cold hardy gene expression. For example, *J.* × *intermedia* (50:50 *J. regia*: *J. nigra*) had lower gene expression than *J. nigra* and much more than pure *J. regia*. Our BC1 (75:25 *J. regia*: *J. nigra*) had lower gene expression than *J.* × *intermedia* but higher expression than pure *J. regia* as well. Some cold hardy gene expression studies reported that F1 hybrids of *Ocimum kilimandscharicum* (Camphor Basil) showed higher expression levels than pure species (Dhawan et al., 2016). Interspecific hybrid *Pinus* spp. with a genetic background composed of 75–87% cold hardy species exhibited greater levels of cold hardiness than others comprised of more cold-sensitive species (Lu et al., 2007). Assessment of cold hardiness using molecular markers represents a significant advancement over long-term (years to decades) field observations and even EL (Joosen et al., 2006). These DREB genes and their associated SNP’s that we have discovered can now serve as a marker assisted selection system for breeding cold hardiness into *Juglans*.

### Phylogenetic Analyses

Whole genome data indicated that the most cold hardy species (*J. cinerea)* sorted with the East Asian species rather than other native *Juglans* species of United States (Figures 5A,B). The *matK* and ITS results from Stanford et al. (2000), also indicated that *J. cinerea* (Trachycaryon) sorted closely with Asian species (*J. regia*, Dioscaryon; *J. mandshurica*, *J. ailantifolia*; Cardiocaryon). Analyses of chloroplast data sorted *J. cinerea* with North American species (Figures 5A,B). These data lend support to the Stanford et al. (2000) hypothesis that *J. cinerea* moved from North America to Eurasia over geologic time following a warming trend during the mid-Oligocene.

Using DREB-like genes, cold hardy *J. cinerea* and *J.* × *bixbi* sorted separately. Other F1 hybrids sorted with *J. nigra* and *J. major* but could not be sorted according to geological history (Figures 5C,D). Protein analyses suggest that proteins coding for cold tolerance genes differed among *Juglans* species. Early research indicated that different proteins within the same species could evolve at dramatically different rates (Zuckerkandl and Pauling, 1965; Wang et al., 2004). This theory was supported by our results in which three *J. regia* IN all sorted to different groups according to DREB gene sequence. Interestingly, *J. regia 3* IN, the hardiest Persian walnut variety in the study, sorted adjacent to the cold hardy *J. mandshurica* (Figure 5D). Stevens et al. (2018) reported that extra copies of a specific gene were due to assembly and demonstrated high heterozygosity, while Martínez-García et al. (2016) highlighted a PPO that may be differentially expressed in a wide range of tissues. Cold hardy genes may have numerous, slightly different copies throughout the genome that work as part of an uncharacterized network; however, further study is required to understand how these genes vary within species.

### *Juglans* Genome Signature of Selection

The signature of selection reveals that cold hardy genes are under strong positive selection in interspecific F1 hybrids compared to *J. regia* from United States, which displayed negative Tajima’s *D* values. Positive selection shows genetic adaptation of species or hybrid in response to abiotic/biotic interactions (Bonhomme et al., 2015). Most hybrid Tajima’s *D* values were ≥1, a rarity unlike the other *Juglans* pools (Table 3 and Supplementary Table 1). *J. regia* was sought out for domestication thousands of years as a high-quality food source (Mercuri et al., 2013). This may explain much of the reduced genetic diversity in the species today. Screens of polymorphic SSR markers in *J. regia* cultivated in the Midwest showed evidence of a genetic bottleneck and severely reduced genetic diversity compared to European Carpathian samples (Ebrahimi et al., 2017). A longitudinal trend of genetic variability was reported in Eurasian *J. regia* populations Pollegioni et al. (2017). These authors highlighted the noticeable loss of allelic richness and heterozygosity from Eastern to Western Europe and the recent reduction in effective population size compared with Asian samples.

In our study, the amount of nucleotide diversity in *J. regia* pools was lower than other *Juglans* species based on cold hardiness genes (Table 3 and Supplementary Table 1) and whole genome analysis (Supplementary Table 2). New studies based on whole genome sequencing (Stevens et al., 2018) revealed that nucleotide diversity in *J. regia* (0.0056) is lower than *J. nigra* (0.0096) and *J. microcarpa* (0.0089), but higher than *J. hindsii* (0.0016). Other studies have noted that allelic numbers and heterozygosity in *J. nigra* were greater than wild *J. regia* populations (Robichaud et al., 2006; Pollegioni et al., 2017). Forward selected seed orchard parents of *J. nigra* displayed fewer alleles than wild selected parents but still maintained high heterozygosity values (Ebrahimi et al., 2018). A recent whole genome study with Chinese chestnut (*Castanea mollissima*) likewise showed that domesticated orchard cultivars were less diverse than wild trees (LaBonte et al., 2018). Studies with hybrid varieties are expected to reveal greater genetic diversity; however, heterozygosity values obtained in this work, although high, were only slightly higher than *J. nigra, J. ailantifolia*, and *J. mandshurica* populations.

## Conclusion

Very little winterkill damage was observed among the *Juglans* species studied from 2003–2013. Most damage resulted from extremely cold temperatures in February 2014. We quantitatively illustrated the correlations between weather, physiological data, and expression of cold hardy genes using whole genome analyses to detect potential markers for assessing cold hardiness in *Juglans* species. Our results indicated that molecular and morpho-physiological data were highly correlated. In addition to morpho-physiology and molecular markers, we employed selective sweep to filter *Juglans* species based on genes related to cold hardiness into different whole genome pools. Should these genes be conserved in other species, these findings may be applicable to more tree genre and species that vary in cold hardiness. All analyses confirmed that cold hardy (*J. nigra* or *J. cinerea*) crossed with cold sensitive (*J. regia* and *J. hindsii*) interspecific F1 hybrids have higher cold hardy gene expression levels than all *J. regia* and the other cold sensitive species examined. As sequencing costs decline, utilization of RNA-sequencing and genome-wide association studies (GWAS) with these genes and their SNP’s should be useful for marker assisted selection of *Juglans* species for cold hardiness.

## Data Availability Statement

The genome sequencing data associated with this study has been deposited in the Hardwood Genomic Project database, and can be accessed using the following links: https://www. hardwoodgenomics.org/bio_data/3641871, https://www.hardwo odgenomics.org/bio_data/3958579.

## Author Contributions

AE designed the project, analyzed the data, and wrote the original draft manuscript. SL contributed to interpretation and visualization of the results and revised the manuscript. JM assisted with collecting field data and providing material for electrolyte leakage and gene expression analysis and revised the manuscript. DJ contributed to interpretation of the results and revised the manuscript. All authors have read and approved the submitted version.

## Conflict of Interest

The authors declare that the research was conducted in the absence of any commercial or financial relationships that could be construed as a potential conflict of interest.

## Footnotes

1 https://htirc.org/

2 https://www.infga.org/

3 http://frodo.wi.mit.edu/primer3/

4 https://www.ncbi.nlm.nih.gov/

5 https://www.purdue.edu/hla/sites/genomics/

6 https://omictools.com/aaf-tool

7 https://www.uniprot.org/blast/

8 http://vcftools.sourceforge.net/

## References

[B1] Aitken S. N. Bemmels J. B. (2016). Time to get moving: assisted gene flow of forest trees. *Evol. Appl.* 9 271–290. 10.1111/eva.12293 27087852 PMC4780373

[B2] Aslamarz A. A. Vahdati K. Hassani D. Rahemi M. Mohammadi N. Leslie C. (2011). Cold hardiness and its relationship with proline content in Persian walnut. *Eur. J. Hort. Sci.* 76 84–90.

[B3] Bai W. N. Yan P. C. Zhang B. W. Woeste K. E. Lin K. Zhang D. Y. (2018). Demographically idiosyncratic responses to climate change and rapid Pleistocene diversification of the walnut genus *Juglans* (Juglandaceae) revealed by whole−genome sequences. *New Phytol.* 217 1726–1736. 10.1111/nph.14917 29178135

[B4] Benedict C. J. Skinner S. Meng R. Chang Y. Bhalerao R. Huner N. P. (2006). The CBF1-dependent low temperature signalling pathway, regulon and increase in freeze tolerance are conserved in *Populus* spp. *Plant Cell Environ.* 29 1259–1272. 10.1111/j.1365-3040.2006.01505.x 17080948

[B5] Boc A. Diallo A. B. Makarenkov V. (2012). T-REX: a web server for inferring, validating and visualizing phylogenetic trees and networks. *Nucleic Acids Res.* 40 573–579. 10.1093/nar/gks485 22675075 PMC3394261

[B6] Bolger A. M. Lohse M. Usadel B. (2014). Trimmomatic: a flexible trimmer for Illumina sequence data. *Bioinformatics* 30 2114–2120. 10.1093/bioinformatics/btu170 24695404 PMC4103590

[B7] Bonhomme M. Boitard S. San Clemente H. Dumas B. Young N. Jacquet C. (2015). Genomic signature of selective sweeps illuminates adaptation of *Medicago truncatula* to root-associated microorganisms. *Mol. Biol. Evol.* 32 2097–2110. 10.1093/molbev/msv092 25901015 PMC4833077

[B8] Buchfink B. Xie C. Huson D. H. (2015). Fast and sensitive protein alignment using DIAMOND. *Nat. Methods* 12 59–60. 10.1038/nmeth.3176 25402007

[B9] Charrier G. Bonhomme M. Lacointe A. Améglio T. (2011). Are budburst dates, dormancy and cold acclimation in walnut trees (*Juglans regia* L.) under mainly genotypic or environmental control? *Int. J. Biomet.* 55 763–774. 10.1007/s00484-011-0470-1 21805380

[B10] Charrier G. Poirier M. Bonhomme M. Lacointe A. Améglio T. (2013). Frost hardiness in walnut trees (*Juglans regia* L.): how to link physiology and modeling. *Tree Physiol.* 33 1229–1241. 10.1093/treephys/tpt090 24271086

[B11] Clair J. B. (2006). Genetic variation in fall cold hardiness in coastal Douglas-fir in western Oregon and Washington. *Botany* 84 1110–1121. 10.1139/b06-084

[B12] Demesure B. (1996). “Conservation of genetic resources of noble hardwoods in France: overview,” in *Noble Hardwoods Network. Report of the First Meeting, Escherode, Germany*, eds Turok J. Eriksson G. Kleinschmit J. Canger S. (Rome: International Plant Genetic Resources Institute), 9–11.

[B13] Dhawan S. S. Shukla P. Gupta P. Lal R. K. (2016). A cold-tolerant evergreen interspecific hybrid of *Ocimum kilimandscharicum* and *Ocimum basilicum*: analyzing trichomes and molecular variations. *Protoplasma* 253 845–855. 10.1007/s00709-015-0847-9 26156173

[B14] Doyle J. J. Doyle J. L. (1987). A rapid DNA isolation procedure for small quantities of fresh leaf tissue. *Phytochem. Bull.* 19 11–15.

[B15] Earnshaw M. J. (1993). *Stress Indicators: Electrolyte Leakage. Methods in Comparative Plant Ecology.* London: Chapman & Hall, 152–154.

[B16] Ebrahimi A. Lawson S. S. Frank G. S. Coggeshall M. V. Woeste K. E. McKenna J. R. (2018). Pollen flow and paternity in an isolated and non-isolated black walnut (*Juglans nigra* L.) *timber seed orchard*. *PLoS One* 13:e0207861. 10.1371/journal.pone.0207861 30513103 PMC6279045

[B17] Ebrahimi A. Zarei A. McKenna J. R. Bujdoso G. Woeste K. E. (2017). Genetic diversity of Persian walnut (*Juglans regia*) in the cold-temperate zone of the United States and Europe. *Sci. Hortic.* 220 36–41. 10.1016/j.scienta.2017.03.030

[B18] Fan H. Ives A. R. Surget-Groba Y. Cannon C. H. (2015). An assembly and alignment-free method of phylogeny reconstruction from next-generation sequencing data. *BMC Genom.* 16:522. 10.1186/s12864-015-1647-5 26169061 PMC4501066

[B19] George M. F. Hong S. G. Burke M. J. (1977). Cold hardiness and deep supercooling of hardwoods: its occurrence in provenance collections of red oak, yellow birch, black walnut, and black cherry. *Ecology* 58 674–680. 10.2307/1939018

[B20] Gilmour S. J. Zarka D. G. Stockinger E. J. Salazar M. P. Houghton J. M. Thomashow M. F. (1998). Low temperature regulation of the *Arabidopsis* CBF family of AP2 transcriptional activators as an early step in cold-induced COR gene expression. *Plant J.* 16 433–442. 10.1046/j.1365-313x.1998.00310.x 9881163

[B21] Grimo E. (1979). “Carpathian (Persian) walnuts,” in *Nut Tree Culture in North America*, ed. Jaynes R. A. (Hamden, CT: Northern Nut Growers Association), 74–83.

[B22] Guàrdia M. Díaz R. Savé R. Aletà N. (2013). Autumn frost resistance on several walnut species: methods comparison and impact of leaf fall. *For. Sci.* 59 559–565. 10.5849/forsci.12-094

[B23] Hamilton J. A. Lexer C. Aitken S. N. (2013). Genomic and phenotypic architecture of a spruce hybrid zone (*Picea sitchensis*× *P. glauca)*. *Mol. Ecol.* 22 827–841. 10.1111/mec.12007 22967172

[B24] Hu Y. Woeste K. E. Zhao P. (2017). Completion of the chloroplast genomes of five Chinese *Juglans* and their contribution to chloroplast phylogeny. *Front. Plant Sci.* 7:1955. 10.3389/fpls.2016.01955 28111577 PMC5216037

[B25] Hyung L. J. Yu D. J. Kim S. J. Choi D. Lee H. J. (2012). Intraspecies differences in cold hardiness, carbohydrate content and β-amylase gene expression of *Vaccinium corymbosum* during cold acclimation and deacclimation. *Tree Physiol.* 32 1533–1540. 10.1093/treephys/tps102 23135736

[B26] Jacobs D. F. Wilson B. C. Ross-Davis A. L. Davis A. S. (2008). Cold hardiness and transplant response of *Juglans nigra* seedlings subjected to alternative storage regimes. *Ann. Forest. Sci.* 65 606–606. 10.1051/forest:2008036

[B27] Joosen R. V. Lammers M. Balk P. A. Brønnum P. Konings M. C. Perks M. (2006). Correlating gene expression to physiological parameters and environmental conditions during cold acclimation of *Pinus sylvestris*, identification of molecular markers using cDNA microarrays. *Tree Physiol.* 26 1297–1313. 10.1093/treephys/26.10.1297 16815832

[B28] LaBonte N. R. Zhao P. Woeste K. (2018). Signatures of selection in the genomes of Chinese chestnut (*Castanea mollissima* Blume): the roots of nut tree domestication. *Front. Plant Sci.* 9:810. 10.3389/fpls.2018.00810 29988533 PMC6026767

[B29] Langmead B. Trapnell C. Pop M. Salzberg S. L. (2009). Ultrafast and memory-efficient alignment of short DNA sequences to the human genome. *Genome Biol.* 10:R25. 10.1186/gb-2009-10-3-r25 19261174 PMC2690996

[B30] Livak K. J. Schmittgen T. D. (2001). Analysis of relative gene expression data using real-time quantitative PCR and the 22DDCT method. *Methods* 25 402–408. 10.1006/meth.2001.1262 11846609

[B31] Lu P. Colombo S. J. Sinclair R. W. (2007). Cold hardiness of interspecific hybrids between *Pinus strobus* and *P. wallichiana* measured by post-freezing needle electrolyte leakage. *Tree Physiol.* 27 243–250. 10.1093/treephys/27.2.243 17241966

[B32] Luo R. Liu B. Xie Y. Li Z. Huang W. Yuan J. (2012). SOAPdenovo2: an empirically improved memory-efficient short-read de novo assembler. *Gigascience* 1:18. 10.1186/2047-217X-1-18 23587118 PMC3626529

[B33] Lyons J. M. Graham D. Raison J. K. (1979). “The plant membrane in response to low temperature: an overview,” in *Low Temperature Stress in Crop Plant*, eds LYONS J. M. GRAHAM D. RAISON J. K. (New York, NY: Academic Press), 4–24.

[B34] Manning W. E. (1978). The classification within the Juglandaceae. *Ann. Mo. Bot. Gard.* 65 1058–1087.

[B35] Marrano A. Britton M. Zaini P. A. Zimin A. Workman R. Puiu D. (2019). High-quality chromosome-scale assembly of the walnut (*Juglans regia* L) reference genome. *bioRxiv* [Preprint]. 10.1101/809798x PMC7238675 32432329

[B36] Martínez-García P. J. Crepeau M. W. Puiu D. Gonzalez−Ibeas D. Whalen J. Stevens K. A. (2016). The walnut (*Juglans regia*) genome sequence reveals diversity in genes coding for the biosynthesis of non−structural polyphenols. *Plant J.* 87 507–532. 10.1111/tpj.13207 27145194

[B37] McKay H. M. (1992). Electrolyte leakage from fine roots of conifer seedlings: a rapid index of plant vitality following cold storage. *Can. J. For. Res.* 22 1371–1377. 10.1139/x92-182

[B38] McKay J. W. (1965). Progress in black x Persian walnut breeding. *Annu. Rep. North Nut Growers Assoc.* 56 76–80.

[B39] McKenna A. Hanna M. Banks E. Sivachenko A. Cibulskis K. Kernytsky A. (2010). The genome analysis toolkit: a mapreduce framework for analyzing next-generation DNA sequencing data. *Genome Res.* 20 1297–1303. 10.1101/gr.107524.110 20644199 PMC2928508

[B40] Mercuri A. M. Bandini M. M. Florenzano A. Montecchi M. C. Rattighieri E. O. (2013). *Juglans* and *Castanea*: the OJC group as pollen evidence of the development of human-induced environments in the Italian peninsula. *Quat. Int.* 303 24–42. 10.1016/j.quaint.2013.01.005

[B41] Mitra S. K. Rathore S. D. Bose T. K. (1991). *Walnut. Temperate Fruits*, Vol. 27 Kolkata: Horticulture and Allied Publishers, 377–414.

[B42] Patterson B. D. Murata T. Graham D. (1976). Electrolyte leakage induced by chilling in *Passiflora* species tolerant to different climates. *Func. Plant Biol.* 3 435–442.

[B43] Poirier M. Bodet C. Ploquin S. Saint-Joanis B. Lacointe A. Améglio T. (2004). “Walnut cultivar performance of cold resistance in south central France,” in *Proceedings of the Fifth International Walnut Symposium*, Sorrento, Vol. 705 281–285. 10.17660/actahortic.2005.705.35

[B44] Pollegioni P. Woeste K. Chiocchini F. Del Lungo S. Ciolfi M. Olimpieri I. (2017). Rethinking the history of common walnut (*Juglans regia* L.) in Europe: its origins and human interactions. *PLoS One* 12:e0172541. 10.1371/journal.pone.0172541 28257470 PMC5336217

[B45] Potts D. T. (2018). Arboriculture in ancient Iran: walnut (*Juglans regia*), plane (*Platanus orientalis*) and the “Radde dictum”. *Dabir* 6 101–109.

[B46] Ramos D. E. (1997). *Walnut Production Manual*, Vol. 3373 Oakland, CA: UCANR Publications.

[B47] Robichaud R. L. Glaubitz J. C. Rhodes O. E. Woeste K. (2006). A robust set of black walnut microsatellites for parentage and clonal identification. *New Forest.* 32 179–196. 10.1007/s11056-005-5961-7

[B48] Sakai A. Weiser C. J. (1973). Freezing resistance of trees in North America with reference to tree regions. *Ecology* 54 118–126. 10.2307/1934380

[B49] Schaberg P. G. DeHayes D. H. (2000). “Physiological and environmental causes of freezing injury in red spruce,” in *Responses of Northern US Forests to Environmental Change*, eds Robert M. A. Richard B. A. John H. (New York, NY: Springer), 181–227. 10.1007/978-1-4612-1256-0_6

[B50] Stanford A. M. Harden R. Parks C. R. (2000). Phylogeny and biogeography of *Juglans* (Juglandaceae) based on matK and ITS sequence data. *Am. J. Bot.* 87 872–882. 10.2307/2656895 10860918

[B51] Stanke M. Keller O. Gunduz I. Hayes A. Waack S. Morgenstern B. (2006). AUGUSTUS: ab initio prediction of alternative transcripts. *Nucleic Acids Res.* 34 435–439. 16845043 10.1093/nar/gkl200 PMC1538822

[B52] Steel R. G. Torrie J. H. (1980). *Principles and Procedures of Statistics, a Biometrical Approach*, 2nd Edn Auckland: McGraw-Hill Kogakusha Ltd.

[B53] Stevens K. A. Woeste K. Chakraborty S. Crepeau M. W. Leslie C. A. Martínez-García P. J. (2018). Genomic variation among and within six *Juglans* species. *G3-Genes Genom Genet*. 8 2153–2165. 10.1534/g3.118.200030 PMC6027890 29792315

[B54] Sutinen M. L. Palta J. P. Reich P. B. (1992). Seasonal differences in freezing stress resistance of needles of *Pinus nigra* and *Pinus resinosa*: evaluation of the electrolyte leakage method. *Tree Physiol.* 11 241–254. 10.1093/treephys/11.3.241 14969949

[B55] Tamura K. Dudley J. Nei M. Kumar S. (2007). MEGA4: molecular evolutionary genetics analysis (MEGA) software version 4.0. *Mol. Boil. Evo.* 24 1596–1599. 10.1093/molbev/msm092 17488738

[B56] Thornton K. R. Jensen J. D. (2007). Controlling the false-positive rate in multilocus genome scans for selection. *Genetics* 175 737–750. 10.1534/genetics.106.064642 17110489 PMC1800626

[B57] Wang G. Kong H. Sun Y. Zhang X. Zhang W. Altman N. (2004). Genome-wide analysis of the cyclin family in *Arabidopsis* and comparative phylogenetic analysis of plant cyclin-like proteins. *Plant Physiol.* 135 1084–1099. 10.1104/pp.104.040436 15208425 PMC514142

[B58] Weiser C. J. (1970). Cold resistance and injury in woody plants: knowledge of hardy plant adaptations to freezing stress may help us to reduce winter damage. *Science* 169 1269–1278. 10.1126/science.169.3952.1269 17772511

[B59] Wisniewski M. Nassuth A. Teulières C. Marque C. Rowland J. Cao P. B. (2014). Genomics of cold hardiness in woody plants. *Crit. Rev. Plant Sci.* 33 92–124.

[B60] Wisniewski M. Norelli J. Artlip T. (2015). Overexpression of a peach CBF gene in apple: a model for understanding the integration of growth, dormancy, and cold hardiness in woody plants. *Front. Plant Sci.* 6:85. 10.3389/fpls.2015.00085 25774159 PMC4343015

[B61] Wysoker A. Tibbetts K. Fennell T. (2013). *Picard Tools Version 1.90.* Available online at: http://picard.sourceforge.net (accessesd April 22, 2013).

[B62] Zarei A. Zamani Z. Mousavi A. Fatahi R. Alavijeh M. K. Dehsara B. (2012). An effective protocol for isolation of high-quality RNA from pomegranate seeds. *Asian Aust. J. Plant Sci. Biotechnol.* 6 32–37.

[B63] Zuckerkandl E. Pauling L. (1965). “Evolutionary divergence and convergence in proteins,” in *Evolving Genes and Proteins*, eds Bryson V. Vogel (New York: Academic Press), 97–166. 10.1016/b978-1-4832-2734-4.50017-6

