# Supplementary material for: Morpho-Physiological and Genomic Evaluation of Juglans Species Reveals Regional Maladaptation to Cold Stress
Source: Front Plant Sci. 2020 Mar 10;11:229. doi: 10.3389/fpls.2020.00229 (PMC7077431; doi:10.3389/fpls.2020.00229)
Supplement: Supplementary file 1 [file Data_Sheet_1.docx]

**Appendix A:** *J. regia* samples, DBH, winter kill, provider and germplasm collection used in this study. ^$^; *J. nigra* (black walnut) used as a rootstock for grafted seedling; Sdlg; seedling.

| Cultivar / Genotype | Age | DBH (cm) | Winter kill | Stock^$^ Type | Species | Provider | Germplasm |
| --- | --- | --- | --- | --- | --- | --- | --- |
| Franquette | 12 | 3.81 | 5.0 ±1 | Graft | *J.regia* | Ca, USA | HTIRC |
| Fernette | 12 | 3.81 | 5.0 ±1 | Graft | *J.regia* | Ca, USA | HTIRC |
| Lozeronne | 11 | 4.31 | 4.8 ±1 | Graft | *J.regia* | Ca, USA | HTIRC |
| Idaho | 12 | 4.82 | 3.0 ±1 | Graft | *J.regia* | ID, USA | HTIRC |
| Ziegler | 11 | 7.62 | 2.5 ±1 | Graft | *J.regia* | IN, USA | HTIRC |
| # 693 | 11 | 7.60 | 2.0 ±1 | Graft | *J.regia* | IN, USA | HTIRC |
| # 694 | 11 | 7.62 | 2.0 ±1 | Graft | *J.regia* | IN, USA | HTIRC |
| # 695 | 11 | 6.6 | 2.0 ±1 | Graft | *J.regia* | IN, USA | HTIRC |
| #03-693p-1 | 11 | 11.9 | 3.0 ±1 | Sdlg. | *J.regia* | IN, USA | HTIRC |
| #03-693p-9 | 11 | 4.82 | 3.0 ±1 | Sdlg. | *J.regia* | IN, USA | HTIRC |
| #03-694p-2 | 11 | 7.11 | 2.0 ±1 | Sdlg. | *J.regia* | IN, USA | HTIRC |
| #03-695p-4 | 11 | 3.81 | 2.9 ±1 | Sdlg. | *J.regia* | IN, USA | HTIRC |
| #03-695p-8 | 11 | 4.82 | 2.0 ±1 | Sdlg. | *J.regia* | IN, USA | HTIRC |
| Kosovo | 10 | 4.31 | 2.0 ±1 | Graft | *J.regia* | Serbia | HTIRC |
| Behr | 60 | 55.88 | 2.2 ±1 | Sdlg. | *J.regia* | IN, USA | HTIRC |
| Summer's Sdlg | 10 | 3.55 | 2.3 ±1 | Graft | *J.regia* | MI, USA | INFGA |
| Hansen Sdlg | 8 | 3.04 | 2.1 ±1 | Graft | *J.regia* | IL, USA | INFGA |
| #02-3-3 | 8 | 3.30 | 2.5 ±1 | Sdlg. | *J.regia* | Russia | INFGA |
| Lake | 8 | 2.54 | 1.9 ±1 | Graft | *J.regia* | MO, USA | INFGA |
| Meridian St. | 40 | 40.64 | 2.0 ±1 | Sdlg. | *J.regia* | IN, USA | INFGA |
| SW Hort Farm | 50 | 50.8 | 2.0 ±1 | Sdlg. | *J.regia* | IN, USA | INFGA |
| Timber Type | 40 | 38.1 | 2.5 ±1 | Graft | *J.regia* | IN, USA | INFGA |
| Univ. Farms | 28 | 33.02 | 2.0 ±1 | Sdlg. | *J.regia* | IN, USA | INFGA |
| Yeager Road | 40 | 45.72 | 2.2 ±1 | Sdlg. | *J.regia* | IN, USA | INFGA |
| Lapaz Tree | 75 | 34 | 2.0 ±1 | Sdlg. | *J.regia* | IN, USA | INFGA |
| Besque-1 | 15 | 17.78 | 1.5 ±1 | Sdlg. | *J.regia* | IN, USA | INFGA |
| Besque-2 | 15 | 17.78 | 2.0 ±1 | Sdlg. | *J.regia* | IN, USA | INFGA |
| Besque-3 | 40 | 63.5 | 2.0 ±1 | Sdlg. | *J.regia* | IN, USA | INFGA |
| King-1 | 25 | 25.4 | 2.5 ±1 | Sdlg. | *J.regia* | IN, USA | INFGA |
| King-2 | 25 | 27.94 | 2.5 ±1 | Sdlg. | *J.regia* | IN, USA | INFGA |
| King-3 | 25 | 33.02 | 2.5 ±1 | Sdlg. | *J.regia* | IN, USA | INFGA |
| King-4 | 25 | 25.4 | 2.0 ±1 | Sdlg. | *J.regia* | IN, USA | INFGA |
| Olsen tree-1 | 45 | 48.26 | 2.2 ±1 | Sdlg. | *J.regia* | IN, USA | INFGA |
| Olsen tree-2 | 45 | 53.34 | 2.5 ±1 | Sdlg. | *J.regia* | IN, USA | INFGA |
| Lhuppert tree | 25 | 25.4 | 2.0 ±1 | Sdlg. | *J.regia* | IN, USA | INFGA |
| 100 w tree | 45 | 55.88 | 2.0 ±1 | Sdlg. | *J.regia* | IN, USA | INFGA |
| Oran Barton-1 | 95 | 91.44 | 2.0 ±1 | Sdlg. | *J.regia* | IN, USA | INFGA |
| Oran Barton-2 | 15 | 15.24 | 2.0 ±1 | Graft | *J.regia* | IN, USA | INFGA |
| Oran Barton-3 | 15 | 17.78 | 2.0 ±1 | Graft | *J.regia* | IN, USA | INFGA |
| Kaiser Persian | 15 | 20.32 | 2.0 ±1 | Graft | *J.regia* | IN, USA | INFGA |
| Lake × Hansen | 15 | 15.24 | 2.0 ±1 | Graft | J.regia | IN, USA | INFGA |

**Appendix B:** Average yearly temperature. Average temperatures from 2002–2016 in Lafayette, IN.


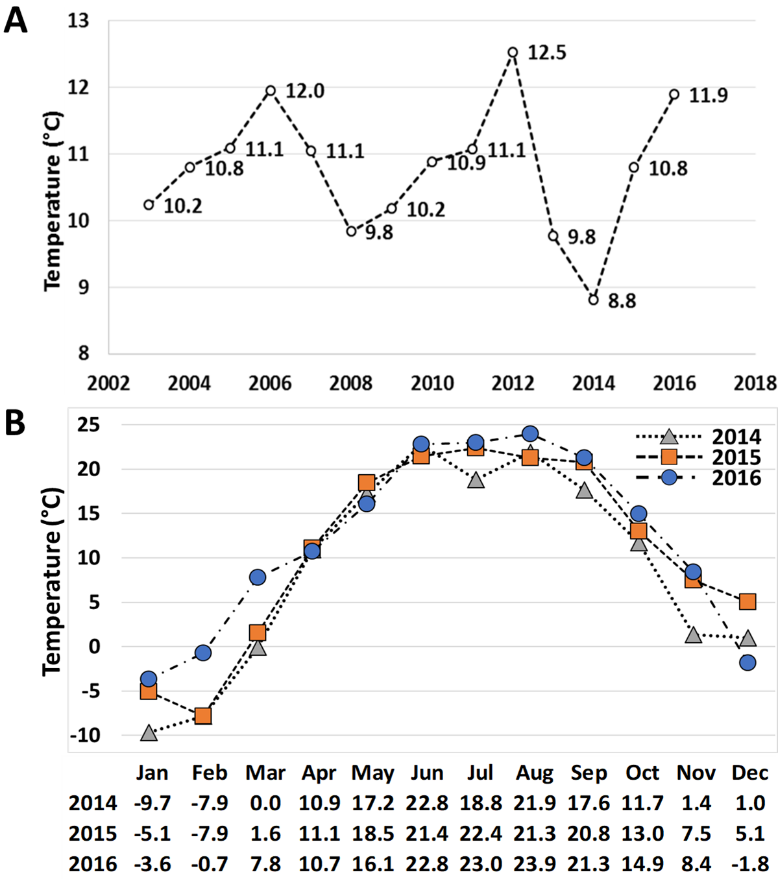


**Appendix C**. Mean (+ SE) winterkill (rated; 1-5), Height (cm), and DBH (cm) of *Juglans* species. Genotypes information listed in **Table 1**. Winter kill rated based on the observation of whole trees and 5-10 twigs evaluated for each individual. *J. regia_CA:* samples provided from California*;* *J. regia_IN:* samples provided from Indiana, HTIRC*;* *J. regia_NG:* samples provided from Indiana Nut Growers*;* Non-significant differences among treatments (α = 0.05).

| **Species** | **Winter Kill** | **Height (cm)** | **DBH (cm)** |
| --- | --- | --- | --- |
| *J. cinerea* | 1 (±0.6) d | 512.5 (±0.6) c | 7.11 (±1.3) bc |
| *J. nigra* | 1 (±0.08) d | 604.4 (±2) b | 8.63 (±0.52) b |
| *J. × bixbi* | 1 (±0 d) d | 542 (±0) c | 7.87 (±0) bc |
| *J.× intermedia* | 1 (±0.9) d | 307 (±1.3) e | 7.11 (±1.3) bc |
| *J. regia_CA* | 5 (±0.4) a | 358.8 (±1.33) e | 4.2 (±0.4) d |
| *J. regia_IN* | 2 (±0.6) c | 454.4 (±1.77) d | 5.08 (±0.3) d |
| *J. regia_NG* | 2 (±0.2) c | 800 (±9.32) a | 38.1 (±0.1) a |
| *J. mandshurica* | 1 (±0.9) d | 346.3 (±1.4) e | 10.9 (±1.9) b |
| *J. ×quadrangulata* | 1 (±0) d | 548.64 (±0) c | 7.62 (±0) bc |
| *J. regia BC1* | 2 (±0) c | 426.72 (±3.5) d | 8.12 (±0) b |
| *J. ailantifolia* | 1 (±0.2) d | 396 (±0.8) de | 4.82 (±0.8) d |
| *J. major* | 5 (±0.9) a | 454 (±1.1) d | 5.08 (±0.8) d |
| *J. nigra × J. major* | 1 (±0) d | 502 (±0) c | 8.3 (±0.0) b |
| *J. nigra × J. hindsii* | 1 (±0.07) d | 413 (±0.95) d | 10.41 (±1.57) b |
| *J. nigra × J. ailantifolia* | 1 (±0.6) d | 731 (±1.9) b | 6.9 (±0.95) c |
| *J. hindsii* | 3 (±0) b | 312 (±2.6) e | 8.4 (±0) b |
| *J. cinerea × J. ailantifolia* | 1 (±0.3) d | 539.5 (±1.5) c | 7.87 (±0.5) bc |

**Appendix D**. Mean (+ SE) of electrolyte leakage damage index at 4 °C, -10 °C, -20 °C and -30 °C of Juglans species. Genotypes information listed in **Table 1**. *J. regia* provided from California and *J. major* evaluated for winter kill data excluded from electrolyte leakage analysis. *J. regia* used in this analysis came from Indiana State. Also *J. major* used in electrolyte leakage analysis provided from Department of Natural Resources (DNR) at Indiana. Non-significant differences among treatments (α = 0.05).

| **Species** | **4 °C** | **-10 °C** | **-20 °C** | **-30 °C** |
| --- | --- | --- | --- | --- |
| *J. cinerea* | 0.53 (±0.0) ns | 3.78 (±0.26) d | 24.29 (±0.48) d | 29.05 (±0.64) h |
| *J. nigra* | 0.48 (±0.0) ns | 3.86 (±0.85) d | 24.75 (±0.96) d | 31.45 (±0.62) h |
| *J. × bixbi* | 1.12 (±0.1) ns | 3.91 (±0.82) d | 26.90 (±0.70) c | 36.22 (±0.149) g |
| *J. × intermedia* | 1.09 (±0.0) ns | 7.25 (±0.65) c | 25.47 (±0.65) c | 45.75 (±0.42) e |
| *J. regia_1 IN* | 1.12 (±0.0) ns | 7.44 (±0.89) c | 27.87 (±1.07) c | 65.10 (±1.19) b |
| *J. regia_2 IN* | 1.15 (±0.4) ns | 13.42 (±1.3) b | 29.32 (±1.04) b | 50.6 (±0.94) d |
| *J. regia_3 IN* | 1.04 (±0.0) ns | 6.07 (±0.83) c | 25.5 (±1.00) c | 46.9 (±0.92) e |
| *J. mandshurica* | 0.32 (±0.0) ns | 6.53 (±0.44) c | 25.50 (±0.88) c | 41.75 (±0.31) f |
| *J. ×quadrangulata* | 0.46 (±0.0) ns | 6.33 (±0.97) c | 27.50 (±0.70) c | 40.78 (±0.23) f |
| *J. regia BC1* | 1.11 (±0.3) ns | 9.65 (±0.96) bc | 25.22 (±0.75) c | 51.55 (±0.46) d |
| *J. ailantifolia* | 0.42 (±0.8) ns | 7.17 (±1.23) c | 28.56 (±0.51) b | 41.5 (±0.67) f |
| *J. major* | 0.89 (±0.0) ns | 19.09 (±1.1) a | 37.19 (±0.70) a | 69.19 (±1.18) a |
| *J. nigra × J.major* | 0.33 (±0.0) ns | 7.78 (±1.69) c | 26.25 (±0.50) c | 47.06 (±0.86) e |
| *J. nigra × J.hindsii* | 0.22 (±0.0) ns | 7.55 (±2.80) c | 26.21 (±0.53) c | 44.45 (±1.33) e |
| *J. nigra × J.ailantifolia* | 0.14 (±0.0) ns | 7.30 (±1.48) c | 30.75 (±0.95) b | 38.84 (±0.75) g |
| *J. hindsii* | 1.37 (±0.1) ns | 6.25 (±1.71) c | 26.75 (±0.96) c | 60.25 (±1.25) c |

**Appendix E**. Mean (+ SE) of gene expression analysis based on primer A of *Juglans* species at 4 °C, -10 °C, -20 °C, -30 °C and -38 °C. Genotypes information listed in **Table 1**. *J. regia* provided from California and *J. major* evaluated for winter kill in the field excluded from gene expression because they killed by frost in 2014. All the *J. regia* used in this analysis came from Indiana State. Also *J. major* used for gene expression analysis provided from Department of Natural Resources (DNR) at Indiana State. Non-significant differences among treatments (α = 0.05).

| Species | **4 ̊C** | **-10 ̊C** | **-20 ̊C** | **-30 ̊C** | **-38 ̊C** |
| --- | --- | --- | --- | --- | --- |
| *J. cinerea* | 0.12 (±0.0) ns | 4.5 (±0.8) a | 5.0 (±0.2) a | 4.5 (±0.6) a | 3 (±0.4) a |
| *J. nigra* | 0.11 (±0.0) ns | 4.0 (±0.1) a | 4.5 (±0.1) a | 4.5 (±0.3) a | 2.8 (±0.8) a |
| *J. ailantifolia* | 0.14 (±0.4) ns | 4 .0 (±0.1) a | 4.2 (±0.3) a | 4.0 (±0.9) a | 2 (±0.9) a |
| *J. mandshuricha* | 0.12 (±0.5) ns | 4.0 (±1.5) a | 3.8 (±0.0) a | 4.5 (±1.2) a | 2.5 (±0.4) a |
| *J. quadrangulata* | 0.10 (±0.0) ns | 4 .0 (±0.9) a | 4.0 (±0.5) a | 4.5 (±1.8) a | 2.5 (±0.6) a |
| *J. × intermedia* | 0.10 (±0.3) ns | 3.5 (±0.3) a | 4.0 (±0.6) a | 4.2 (±0.2) a | 2.6 (±0.8) a |
| *J. × bixbi* | 0.12 (±0.3) ns | 4.0 (±1.5) a | 4.2 (±0.0) a | 4.2 (±0.8) a | 2.8 (±0.0) a |
| *J. regiaBC1* | 0.12 (±0.0) ns | 3.0 (±0.6) ab | 2.8 (±0.3) b | 1.5 (±0.0) b | 1.8 (±0.0) b |
| *J. regia 1* | 0.12 (±0.1) ns | 3.0 (±0.8) ab | 1.0 (±0.4) c | 0.32 (±0.0) c | 0.0 (±0.0) c |
| *J. regia 2* | 0.10 (±0.0) ns | 2.5 (±0.8) b | 1.0 (±0.5) c | 0.22 (±0.6) c | 0.0 (±0.0) c |
| *J. regia 3* | 0.12 (±0.1) ns | 4 .0 (±0.4) a | 3.0 (±0.4) b | 1.8 (±0.4) b | 0.8 (±0.0) b |
| *J. major* | 0.12 (±0.0) ns | 3.5 (±0.3) a | 1.5 (±0.0) c | 0.1 (±0.4) c | 0 .0 (±0.0) c |

**Appendix F**. Mean (+ SE) of gene expression analysis based on primer B of *Juglans species* at 4 °C, -10 °C, -20 °C, -30 °C and -38 °C. Genotypes information listed in **Table 1**. *J. regia* provided from California and *J. major* evaluated for winter kill in the field excluded from gene expression because they killed by frost in 2014. All the *J. regia* used in this analysis came from Indiana State. Also *J. major* used in gene expression analysis provided from Department of Natural Resources (DNR) at Indiana State. Non-significant differences among treatments (α = 0.05).

| Species | **4 ̊C** | **-10 ̊C** | **-20 ̊C** | **-30 ̊C** | **-38 ̊C** |
| --- | --- | --- | --- | --- | --- |
| *J. cinerea* | 0.0 (±0.0) ns | 6.0 (±1) a | 8.0 (±1.5) a | 7.0 (±1.2) a | 3.6 (±0.9) a |
| *J. nigra* | 0.0 (±0.0) ns | 5.0 (±0.2) a | 8.0 (±0.8) a | 6.5 (±0.3) a | 3.5 (±0.8) a |
| *J. ailantifolia* | 0.0 (±0.0) ns | 5.0 (±0.3) a | 8.0 (±0.3) a | 5.5 (±0.9) a | 2.8 (±0.2) a |
| *J. mandshuricha* | 0.08 (±0.0) ns | 5.0 (±0.8) a | 7.5 (±0.0) a | 5.8 (±0.2) a | 3.0 (±0.4) a |
| *J.quadrangulata* | 0.04 (±0.0) ns | 5.0 (±0.4) a | 7.5 (±0.9) a | 6.0 (±0.7) a | 3.0 (±0.5) a |
| *J. x intermedia* | 0.0 (±0.0) ns | 4.5 (±0.3) ab | 8.0 (±0.6) a | 4.5 (±0.1) b | 2.8 (±0.7) a |
| *J. x bixbi* | 0.0 (±0.0) ns | 5.5 (±0.5) a | 8.0 (±0.0) a | 6.5 (±0.6) a | 3.2 (±0.7) a |
| *J. regiaBC1* | 0.1 (±0.0) ns | 4.0 (±0.4) b | 5.0 (±0.5) b | 3.8 (±0.0) c | 1.0 (±0.9) b |
| *J. regia_1* | 0.1 (±0.0) ns | 4.0 (±0.8) b | 5.0 (±0.6) b | 0.9 (±0.0) d | 0.0 (±0) c |
| *J. regia_2* | 0.0 (±0.0) ns | 4.0 (±0.6) b | 5.0 (±0.5) b | 0.5 (±0.5) d | 0.0 (±0) c |
| *J. regia_3* | 0.2 (±0.1) ns | 4.5 (±0.5) ab | 6.0 (±1) b | 3.5 (±0.5) c | 0.8 (±0.5) b |
| *J. major* | 0.0 (±0.0) ns | 5.0 (±1.1) a | 4.5 (±1.5) b | 1 .0 (±0.5) d | 0.0 (±0) c |
